# Assessment of Overuse of Medical Tests and Treatments at US Hospitals Using Medicare Claims

**DOI:** 10.1001/jamanetworkopen.2021.8075

**Published:** 2021-04-27

**Authors:** Kelsey Chalmers, Paula Smith, Judith Garber, Valerie Gopinath, Shannon Brownlee, Aaron L. Schwartz, Adam G. Elshaug, Vikas Saini

**Affiliations:** 1 Lown Institute, Brookline, Massachusetts; 2 Menzies Centre for Health Policy, Sydney School of Public Health, University of Sydney, Sydney, New South Wales, Australia; 3 Department of Medical Ethics and Health Policy, Perelman School of Medicine, The University of Pennsylvania, Philadelphia; 4 Division of General Internal Medicine, Perelman School of Medicine, The University of Pennsylvania, Philadelphia; 5 Center for Health Equity Research and Promotion, Corporal Michael J. Crescenz Veterans Administration Medical Center, Philadelphia, Pennsylvania; 6 Centre for Health Policy, Melbourne School of Population and Global Health, The University of Melbourne, Melbourne, Victoria, Australia; 7 University of Southern California, Brookings Schaeffer Initiative for Health Policy, The Brookings Institution, Washington, DC

## Abstract

**Importance:**

Overuse of health care services exposes patients to unnecessary risk of harm and costs. Distinguishing patterns of overuse among hospitals requires hospital-level measures across multiple services.

**Objective:**

To describe characteristics of hospitals associated with overuse of health care services in the US.

**Design, Setting, and Participants:**

This retrospective cross-sectional analysis used Medicare fee-for-service claims data for beneficiaries older than 65 years from January 1, 2015, to December 31, 2017, with a lookback of 1 year. Inpatient and outpatient services were included, and services offered at specialty and federal hospitals were excluded. Patients were from hospitals with the capacity (based on a claims filter developed for this study) to perform at least 7 of 12 investigated services. Statistical analyses were performed from July 1, 2020, to December 20, 2020.

**Main Outcomes and Measures:**

Outcomes of interest were a composite overuse score ranging from 0 (no overuse of services) to 1 (relatively high overuse of services) and characteristics of hospitals clustered by overuse rates. Twelve published low-value service algorithms were applied to the data to find overuse rates for each hospital, normalized and aggregated to a composite score and then compared across 6 hospital characteristics using multivariable regression. A k-means cluster analysis was used on normalized overuse rates to identify hospital clusters.

**Results:**

The primary analysis was performed on 2415 cohort A hospitals (ie, hospitals with capacity for 7 or more services), which included 1 263 592 patients (mean [SD] age, 72.4 [14] years; 678 549 women [53.7%]; 101 017 191 White patients [80.5%]). Head imaging for syncope was the highest-volume low-value service (377 745 patients [29.9%]), followed by coronary artery stenting for stable coronary disease (199 579 [15.8%]). The mean (SD) composite overuse score was 0.40 (0.10) points. Southern hospitals had a higher mean score than midwestern (difference in means: 0.06 [95% CI, 0.05-0.07] points; *P* < .001), northeast (0.08 [95% CI, 0.06-0.09] points; *P* < .001), and western hospitals (0.08 [95% CI, 0.07-0.10] points; *P* < .001). Nonprofit hospitals had a lower adjusted mean score than for-profit hospitals (−0.03 [95% CI, −0.04 to −0.02] points; *P* < .001). Major teaching hospitals had significantly lower adjusted mean overuse scores vs minor teaching hospitals (difference in means, −0.07 [95% CI, −0.08 to −0.06] points; *P* < .001) and nonteaching hospitals (−0.10 [95% CI, −0.12 to −0.09] points; *P* < .001). Of the 4 clusters identified, 1 was characterized by its low counts of overuse in all services except for spinal fusion; the majority of major teaching hospitals were in this cluster (164 of 223 major teaching hospitals [73.5%]).

**Conclusions and Relevance:**

This cross-sectional study used a novel measurement of hospital-associated overuse; results showed that the highest scores in this Medicare population were associated with nonteaching and for-profit hospitals, particularly in the South.

## Key Points

**Question:**

What hospital characteristics are associated with overuse of health care services in the US?

**Findings:**

In this cross-sectional study of 1 325 256 services performed at 3351 hospitals, we found that hospitals in the South, for-profit hospitals, and nonteaching hospitals were associated with the highest rates of overuse.

**Meaning:**

Variation within specific hospital types and regions may uncover opportunities for targeted interventions to address overuse.

## Teaser

This cross-sectional study uses Medicare fee-for-service claims data to assess overuse of medical tests and treatments in US hospitals.

## Introduction

Overuse is defined as the delivery of tests and procedures that provide little or no clinical benefit, are unlikely to have an impact on clinician decisions, increase health care spending without improving health outcomes, or risk patient harm in excess of potential benefits.^1^ Estimates suggest that overuse contributes $75.7 billion to $101.2 billion to wasted US health care spending annually.^2,3,4^ Studies at the level of physicians, organizations, and hospital referral regions have measured overuse patterns in claims data, including Medicare, Medicaid, and commercially insured populations.^5,6,7,8,9,10^ These results show considerable variation across physician organizations, including within hospital referral regions and across physicians within the same organization, although the included physician demographic characteristics did not explain a substantial amount of such variation.^10^

Although clinicians are responsible for ordering tests and treatments, their practice patterns may be influenced by hospital policies and culture. Hospital-level interventions to reduce overuse exist,^11^ but to measure and compare their success, a hospital-level measure is required. This study offers such a measure, based on the overuse rates of 12 low-value services, and compares rates across hospital regions, ownership type, safety net status, and teaching status. We also use cluster analysis to investigate patterns of overuse and whether these patterns are associated with particular hospital characteristics.

## Methods

### Data Sources

This cross-sectional study used a 100% sample from the Centers for Medicare & Medicaid Services’ (CMS) Chronic Conditions Data Warehouse of Medicare Fee-For-Service data from the Medicare Provider Analysis and Review table, inpatient, outpatient, and carrier claims filed at short-term general or critical access hospitals from January 1, 2015, to December 31, 2017. We excluded Medicare Advantage claims and Kaiser Permanente hospitals dominated by patients with Medicare Advantage, specialty hospitals (hospitals with more than 20% of their inpatient admissions as either orthopedic or cardiac diagnosis-related groups), hospitals not on the 2019 CMS Hospital Compare website,^12^ and federal hospitals. This study was approved and granted a patient waiver of consent by the New England institutional review board because there were minimal risks for participants and the authors had no contact with any individuals in the study. We followed the Strengthening the Reporting of Observational Studies in Epidemiology (STROBE) reporting guideline.^28^

### Overuse Indicators

We selected 13 low-value services from Schwartz et al^5^ and Segal et al^13^ that we agreed were likely to be provided by hospitals. The included services were knee arthroscopy, vertebroplasty, inferior vena cava filter, renal artery stenting, pulmonary artery catheterization in the intensive care unit, hysterectomy, carotid endarterectomy, coronary artery stenting, spinal fusion, electroencephalogram for 2 low-value indications (syncope and headaches), carotid artery imaging, and head imaging.

Our unit of observation was a unique service date per beneficiary. We modified 6 of these overuse indicators after quality checks on the results indicated some potential misclassification of appropriate services as low value. To enhance the specificity, we added additional exclusion criteria not in the original published reports. The details of these updated algorithms are listed in eTable 2 in the Supplement.

Within the Medicare claims data, we converted the *International Statistical Classification of Diseases and Related Health Problems, Tenth Revision (ICD-10)* procedure and diagnosis codes (present in the data after October 2015) to *International Classification of Diseases, Ninth Revision (ICD-9)* using CMS’ general equivalence mapping tables^14^ in order to apply these algorithms, which used ICD-9 codes.

We decided to exclude pulmonary artery catheterization because of its low volume (290 total services in 2015-2017). Our composite overuse score therefore included 12 services.

To avoid labeling hospitals as having no overuse because they could not offer a service (eg, if they lacked the necessary equipment), we created a capacity filter for each service. This filter included hospitals with at least 1 claim per year for services similar to, or using similar facilities as, the low-value service in question (eTable 1 in the Supplement).

There were 3359 hospitals that had capacity to provide at least 1 service. Our primary study population included hospitals with the capacity for 7 or more services (n = 2415, cohort A). We assessed the stability of these findings with a subanalysis on a second cohort of hospitals with the capacity for all services (n = 1350 hospitals, cohort B).

### The Composite Overuse Score

#### Overuse Rates

Calculating a composite score was done in 4 steps: (1) calculating overuse rates for each service, (2) reliability adjustment of these rates for denominator volume, (3) normalizing the range of rates across services, and (4) calculating the weighted sum of these values for each hospital.

Developing an overuse metric from multiple indicators that use different denominators and patient populations presented a challenge. Chalmers et al^15^ described 3 types of denominators for quantifying low-value care: the specified service volume, the volume of patients with a specific condition, or the volume of all patients. We used the total patient volume as the denominator for those services that are low value in most cases (vertebroplasty, knee arthroscopy, renal stenting, and inferior vena cava filter). For the remaining services, where there was some benefit in certain circumstances, we used a service-specific (for the procedures) or diagnosis-specific (for tests and imaging) denominator.

We used an empirical Bayes reliability adjustment on these overuse rates to adjust small-denominator hospitals toward the overall mean.^16^ This adjustment assumes there is a prior distribution of hospital overuse counts and that hospital estimates with small denominators are less reliable than those with larger volumes. For each service, we fit a β distribution to all hospital overuse rates not equal to 0 or 1 in order to obtain a prior distribution of the overuse rates; this was done in R using the fitdistrplus package (R Foundation).^17^ The histograms of all rates and these fitted distributions are shown in eFigure 1 in the Supplement. Using the estimated parameters for each service, α and β, the adjusted rate for hospital *i* was as follows:

*R_adj i_* = (*s_i_* + α)/(*d_i_* + α + β),

where *s_i_* and *d_i_* are the numerator and denominator count for the hospital’s service overuse rate.

We then standardized the adjusted overuse rates from 0 to 1 using minimum-maximum normalization, as the overuse rates varied widely across the services owing to differences in denominator volumes. In order to remove the effect of a small number of hospitals with outlier rates on this rescaling, we first limited the rates to 3 times the SD away from the mean hospital rate for each service by replacing any rates greater or lower than this with the upper and lower bound.

#### Overuse Score Calculation

The overuse score was a sum of the normalized adjusted overuse rates weighted by the total counts of low-value services across all hospitals. This calculation prioritized services with the highest effect (by volume) on patients nationally. For cohort A, we redistributed the weights of any missing (that is, no capacity) services in our composite score calculation.

### Cluster Analysis

To investigate patterns of overuse across the 12 services, we used k-means cluster analysis to group hospitals based on their normalized adjusted overuse rates using scikit-learn software for the Python programming language.^18^ We selected the number of clusters visually using a scree plot and then assigned labels to each cluster based on the apparent patterns across services.

### Hospital Characteristics

We defined the following hospital characteristics for our comparative analysis: safety net, teaching and financial status, size, geographic region, and core-based statistical area. We ranked hospitals by their proportion of patient stays billed as dual eligible and designated the highest 20% as safety net hospitals. We derived the geographic region from the 2010 Census Regions and Divisions of the United States report. The remaining characteristics were defined using the American Hospital Association 2017 data set.^19^ Hospital size was based on bed counts. Designation as a major teaching hospital required membership in the Council of Teaching Hospitals or the Association of American Medical Colleges. Minor teaching hospitals needed only a medical school affiliation as reported to the American Medical Association. For the core-based statistical area, metropolitan areas have 50 000 or more people, micropolitan regions have 10 000 to 50 000 people, and all other areas are considered rural.^20^ Hospitals designated government or nonfederal and nongovernment or not-for-profit were labeled as nonprofit; the remaining category of investor-owned (for-profit) was considered for-profit hospitals. We excluded 8 hospitals with missing American Hospital Association data.

### Statistical Analysis

We used multiple linear regression to report the adjusted composite overuse means for each hospital characteristic level, adjusted for the other hospital characteristics.^21^ We made post-hoc pairwise comparisons of hospital characteristics with Tukey *P* value and CI adjustment. A *P* value of 0.05 was used to indicate significance, and all tests were 2-sided. For the cluster comparison, we compared the proportions of each hospital characteristic within each cluster against its proportion in the entire cohort of hospitals. Because this difference in proportions is largely affected by sample size, we also calculated the Cohen *h* value and reported results where *h* was greater than 0.2.^22^

Claims analysis was performed using SAS Enterprise, version 7.15 HF8 (SAS Institute) on the CMS Virtual Research Data Center, and statistical analyses were performed from July 1, 2020, to December 20, 2020, using Python programming, version 3.7 and R, version 4.0.0 (using the tidyverse, ggplot2, ggridges, and matplotlib packages; R Foundation).^23,24,25,26,27^ The hospital normalized rates, characteristics, and clusters output are available for reference.^35^

## Results

Table 1 reports the patient and hospital characteristics in our sample, and Table 2 reports the observed low-value service counts and the denominator counts for cohorts A and B. There were 1 325 256 low-value services from January 1, 2015, to December 31, 2017, in the entire population (3351 hospitals) with the capacity to perform at least 1 of the 12 services. The primary analysis was performed on 2415 cohort A hospitals (ie, hospitals with capacity for 7 or more services), which included 1 263 592 patients (mean [SD] age, 72.4 [14] years; 678 549 women [53.7%]; 101 017 191 White patients [80.5%]). Head imaging for syncope was the highest-volume low-value service (377 745 [29.9%]), followed by coronary artery stenting for stable coronary disease (199 579 [15.8%]) and carotid artery imaging for syncope (131 236 [10.8%]).

**Table 1.** Patient and Hospital Characteristics in Our Sample

Characteristic	No. (%)^a^		
	All hospitals (N = 3351)	Cohort A hospitals: capacity for 7 or more services (n = 2415)	Cohort B hospitals: capacity for 12 services (n = 1350)
Total low-value services, No.	1 325 256	1 263 592	1 012 489
Patient age, mean (SD), y	73.4 (14)	72.4 (14)	72.3 (14)
Patient sex			
Women, No. (%)	727 566 (54.9)	678 549 (53.7)	539 657 (53.3)
Men, No. (%)	597 690 (45.1)	585 043 (46.3)	472 832 (46.7)
Hospital safety net status			
Non–safety net	2661 (79)	1998 (83)	1204 (89)
Safety net	690 (21)	417 (17)	146 (11)
Hospital ownership			
For profit	542 (16)	444 (18)	250 (19)
Nonprofit	2809 (84)	1971 (82)	1100 (81)
Teaching class			
Major teaching	224 (6.7)	223 (9.2)	216 (16)
Minor teaching	808 (24)	745 (31)	516 (38)
Nonteaching	2319 (69)	1447 (60)	618 (46)
Hospital size			
≥200 beds	1164 (35)	1154 (48)	1013 (75)
6-199 beds	2187 (65)	1261 (52)	337 (25)
Hospital CBSA type			
Urban	2117 (63)	1853 (77)	1257 (93)
Suburban	601 (18)	421 (17)	89 (6.6)
Rural	633 (19)	141 (5.8)	4 (0.3)
Region			
Midwest	995 (30)	638 (26)	338 (25)
Northeast	489 (15)	432 (18)	226 (17)
South	1281 (38)	917 (38)	539 (40)
West	586 (17)	428 (18)	247 (18)

Abbreviation: CBSA, core-based statistical area.

^a^ Values are listed as No. (%) unless otherwise specified.

**Table 2.** The 12 Low-Value Services and Denominator Descriptions, as Well as the Total Low-Value Service Counts and Spread Across Hospitals

Measure	Numerator	Denominator	Cohort A hospitals (n = 2415)^a^		Cohort B hospitals (n = 1350)		Comp. weight (%)
			Total No.	Rate (N/D) per 100	Total No.	Rate per 100	
Procedures							
Knee arthroscopy	Arthroscopic debridement/chondroplasty of the knee with diagnosis of osteoarthritis or chondromalacia in the procedure claim	Patient volume	105 459	0.03	71 296	0.03	8.4
Vertebroplasty	Vertebroplasty for osteoporosis fractures	Patient volume	94 200	0.03	80 429	0.03	7.2
IVC filter	IVC filter	Patient volume	40 916	0.01	35 974	0.01	3.1
Renal artery stenting	Renal artery stenting for hypertension	Patient volume	12 239	0.003	11 207	0.004	0.9
Hysterectomy	Hysterectomy for benign disease	All hysterectomies	97 831	65.4	81 601	62.5	7.6
CEA	Carotid endarterectomy for patients without stroke/TIA	All carotid endarterectomies	47 612	52.4	44 556	52.3	3.6
Coronary stents	Coronary artery stenting for stable heart disease	All coronary artery stents	199 579	24.8	186 550	24.8	15.1
Spinal fusion	Spinal fusion without radicular pain, herniated disc	All spinal fusions	72 258	20.5	65 866	20.4	5.5
Diagnostic tests and imaging							
EEG for syncope	EEG for syncope	All claims with primary diagnosis of syncope (with exclusions)^b^	77 084	3.6	60 988	3.6	5.9
EEG for headache	EEG for headache	All claims with primary diagnosis of headache (with exclusions)	7433	0.38	5886	0.38	0.6
Carotid artery imaging for syncope	Carotid artery imaging for syncope	All claims with primary diagnosis of syncope (with exclusions)^c^	131 236	11.0	96 231	10.6	10.8
Head imaging for syncope	Head imaging for syncope	All claims with primary diagnosis of syncope (with exclusions)^c^	377 745	27.0	271 905	25.3	31.4

Abbreviations: CEA, carotid endarterectomy; comp, composite; EEG, electroencephalogram; IVC, inferior vena cava; N/D, numerator/denominator; TIA, transient ischemic attack.

^a^ Cohort A hospitals had capacity for 7 or more services, whereas cohort B hospitals had capacity for 12 services (excluding pulmonary artery catheterization).

^b^ Syncope defined using *International Classification of Diseases, Ninth Revision (ICD-9)* codes from Segal: 78.02, 99.21, 33.701.

^c^ Syncope defined using *ICD-9* codes from Schwartz: 78.02, 99.21.

Within visits where syncope was the primary diagnosis and facial/head trauma diagnoses were excluded, 377 745 patients (27.0%) received head imaging (interquartile range [IQR], 22.1%-37.8% across hospitals), the highest proportion among the 4 investigated diagnostic services. The overuse rates and their density across all hospitals are shown in eFigure 1 in the Supplement.

For any visit with a percutaneous coronary stent, 24.8% of visits were for a patient with likely stable coronary disease and no unstable angina or acute myocardial infarction (IQR, 13.8%-27.1% across hospitals). Overall 11.0% of patients with syncope had carotid artery imaging (IQR, 7.1%-15.9%).

### Overuse Scores

**Figure 1.**
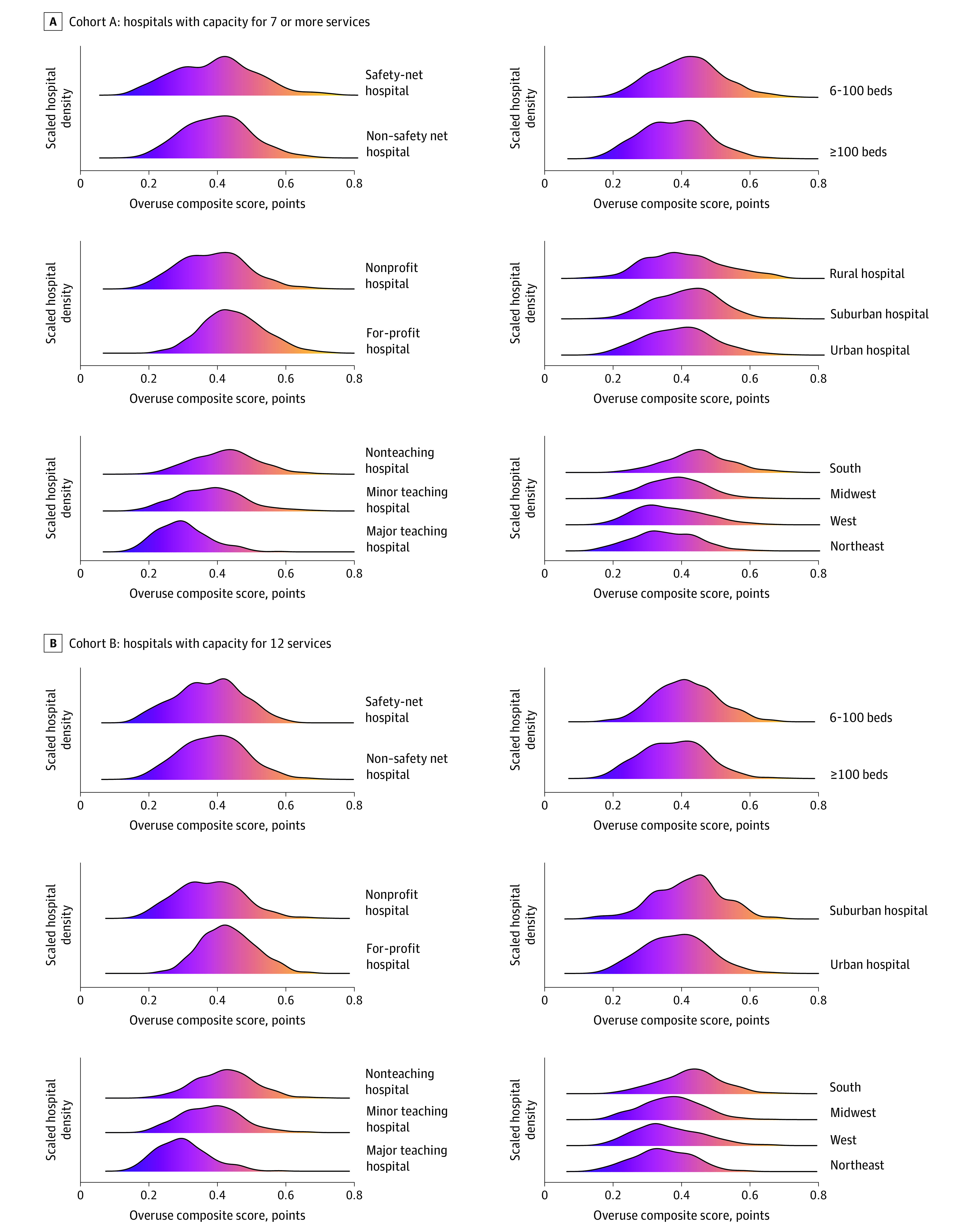
Overuse Composite Scores by Hospital Characteristic A, Density plots of the overuse composite score for hospitals with capacity for 7 or more services (cohort A) in safety and non–safety net hospitals, nonprofit and for-profit hospitals, teaching and nonteaching hospitals, number of beds per hospital, rural, suburban, and urban hospitals, and hospitals based on geographic location. B, Density plots of the overuse composite score for hospitals with capacity for 12 services (cohort B) in safety and non–safety net hospitals, nonprofit and for-profit hospitals, teaching and nonteaching hospitals, number of beds per hospital, rural, suburban, and urban hospitals, and hospitals based on geographic location.

Overuse scores ranged across hospitals from 0.13 to 0.73 points, with a mean (SD) composite overuse score of 0.40 (0.10) points. The distribution of the overuse scores across hospitals is shown in eFigure 2 in the Supplement. Major teaching hospitals had significantly lower adjusted mean overuse scores vs minor teaching hospitals (difference in means, −0.07 [95% CI, −0.08 to −0.06] points; *P* < .001) and nonteaching hospitals (−0.10 [95% CI, −0.12 to −0.09] points; *P* < .001) (Table 3 shows unadjusted and adjusted results). Nonprofit hospitals had a lower adjusted mean score than for-profit hospitals (−0.03 [95% CI, −0.04 to −0.02] points; *P* < .001). There were significant regional differences; southern hospitals had a higher mean score than midwestern (difference in means: 0.06 [95% CI, 0.05-0.07] points; *P* < .001), northeast (0.08 [95% CI, 0.06-0.09] points; *P* < .001), and western hospitals (0.08 [95% CI, 0.07-0.10] points; *P* < .001). Smaller hospitals (<200 beds) had a larger adjusted mean than larger hospitals (0.02 [95% CI, 0.01-0.03] points; *P* < .001). Figure 1 shows the density of these scores by hospital characteristics so readers can visualize these differences across all hospitals.

**Table 3.** Unadjusted and Adjusted Means of the Composite Overuse Score Across Hospitals

Hospital characteristic	Composite scores for cohort A			Composite scores for cohort B		
	Mean (SD)	Adjusted mean (95% CI)^a^	*P* value^b^	Mean (SD)	Adjusted mean (95% CI)	*P* value
Safety net status						
Non–safety net	0.4 (0.1)	0.4 (0.4-0.4)	.32	0.4 (0.1)	0.4 (0.4-0.41)	.06
Safety net	0.4 (0.12)	0.4 (0.39-0.41)		0.39 (0.1)	0.4 (0.39-0.42)	
Ownership type						
For profit	0.45 (0.09)	0.42 (0.41-0.43)	<.001	0.45 (0.08)	0.42 (0.41-0.43)	<.001
Nonprofit	0.39 (0.1)	0.39 (0.39-0.4)		0.39 (0.1)	0.4 (0.39-0.4)	
Teaching class						
Major teaching	0.3 (0.08)	0.32 (0.3-0.33)	<.001	0.31 (0.08)	0.32 (0.31-0.33)	<.001
Minor teaching	0.38 (0.1)	0.39 (0.38-0.39)		0.4 (0.09)	0.4 (0.39-0.41)	
Nonteaching	0.42 (0.1)	0.42 (0.41-0.42)		0.44 (0.09)	0.43 (0.43-0.44)	
Hospital size						
≥200 beds	0.38 (0.1)	0.39 (0.38-0.4)	.02	0.39 (0.1)	0.4 (0.39-0.4)	.23
6-199 beds	0.42 (0.1)	0.41 (0.4-0.41)		0.43 (0.09)	0.41 (0.4-0.42)	
CBSA type						
Urban	0.39 (0.1)	0.4 (0.4-0.41)	.60	0.4 (0.1)	0.4 (0.4-0.41)	.30
Suburban	0.41 (0.09)	0.39 (0.38-0.4)		0.44 (0.1)	0.4 (0.39-0.42)	
Rural	0.41 (0.11)	0.38 (0.37-0.4)		0.41 (0.04)	0.38 (0.29-0.46)	
Region						
Midwest	0.38 (0.09)	0.39 (0.38-0.39)	<.001	0.38 (0.09)	0.39 (0.38-0.4)	<.001
Northeast	0.35 (0.09)	0.37 (0.36-0.37)		0.36 (0.09)	0.38 (0.37-0.39)	
South	0.45 (0.09)	0.44 (0.44-0.45)		0.44 (0.09)	0.43 (0.43-0.44)	
West	0.37 (0.1)	0.36 (0.35-0.37)		0.38 (0.1)	0.37 (0.36-0.38)	

Abbreviation: CBSA, core-based statistical area.

^a^ Adjusted means are based on linear regression using all hospital characteristics.

^b^ Comparison *P* value is from the analysis of variance comparison of the linear regression model and tests whether the hospital characteristic is significant in this model.

### Hospital Clusters

**Figure 2.**
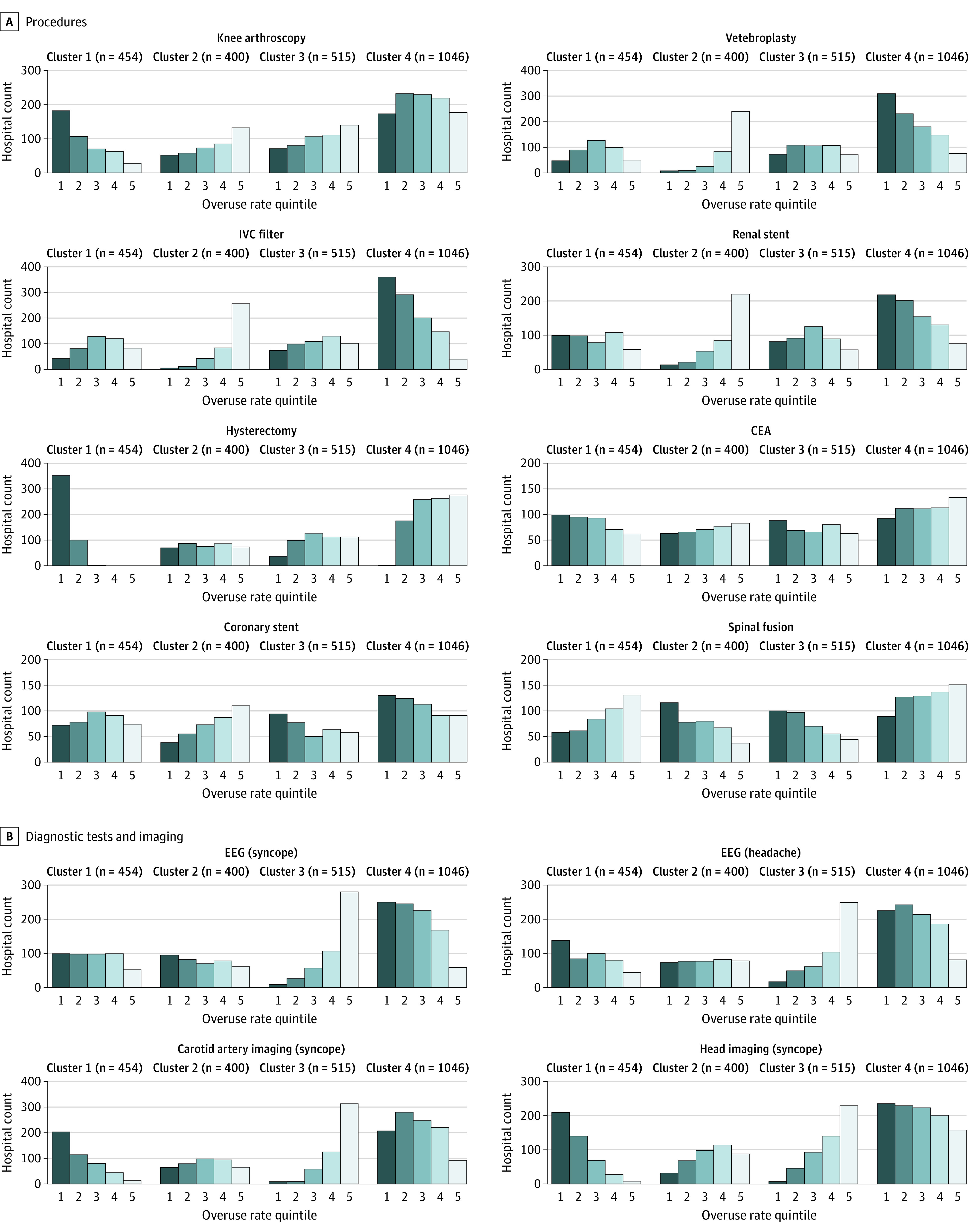
Counts Within Quintiles for 12 Low-Value Services in 4 Identified Hospital Clusters A, Cluster profiles for hospitals with capacity for 7 or more services (cohort A, N = 2415 hospitals) in reference to the following procedures: knee arthroscopy, vertebroplasty, IVC filter, renal stent, hysterectomy, CEA, coronary stent, and spinal fusion. B, Cluster profiles for hospitals with capacity for 7 or more services in reference to the following diagnostic tests and imaging: electroencephalogram (EEG) (syncope), EEG (headache), carotid artery imaging (syncope), and head imaging (syncope). Bars show the counts of quintiles of the normalized overuse hospital rates for each service across the 4 clusters. CEA indicates carotid endarterectomy; IVC, inferior vena cava.

Overuse rates for each service fell into 4 distinct clusters in cohort A (eFigures 3 and 4 in the Supplement show the selection and visualization of these clusters). Figure 2 shows the quintile counts of the rates across these clusters. For each cluster, we report the hospital characteristics with a significantly and largely different (that is, if Cohen *h* > 0.2) proportion within the cluster compared with all hospitals in the cohort (eTable 3 in the Supplement).

Cluster 1 had hospitals with generally low overuse except for spinal fusion. Major teaching hospitals tended to be found in this cluster (41.2% in cluster 1 vs 16.0% overall; *t* statistic, 17.5; *P* < .001; Cohen *h* value, 0.57), as did nonprofit hospitals (92.9% in cluster 1 vs 81.5% overall; *t* statistic, 6.9; *P* < .001; Cohen *h* value, 0.35), and large hospitals (>200 beds) (90.8% in cluster 1 vs 75.0% overall; *t* statistic, 6.9; *P* < .001; Cohen *h* value, 0.50). Cluster 2 showed higher overuse rates across most invasive procedures than the other 3 clusters, and had more for-profit hospitals (35.7% in cluster 2 vs 18.5% overall; *t* statistic, 6.4; *P* < .001; Cohen *h* value, 0.39) and southern hospitals (61.1% in cluster 2 vs 40.0% overall; *t* statistic, 7.6; *P* < .001; Cohen *h* value, 0.43). Cluster 3 hospitals had higher overuse of the 4 diagnostic services compared with other clusters and had a larger share of nonteaching hospitals (59.8% in cluster 3 vs 45.8% overall; *t* statistic, 4.1; *P* < .001; Cohen *h* value, 0.28).

Hospitals in cluster 4 had higher rates of overuse of hysterectomy than other clusters, but lower overuse scores for vertebroplasty, inferior vena cava filters, renal stenting, and the diagnostic services of electroencephalogram and carotid imaging. This group had a higher share of smaller hospitals (40.4% in cluster 4 vs 25.0% overall; *t* statistic, 5.9; *P* < .001; Cohen *h* value, 0.33).

### Results for Cohort B: Hospitals With Capacity for All 12 Services

Cohort B had fewer smaller, safety net and rural hospitals than cohort A. Differences in the mean overuse scores across hospital characteristics were similar to cohort A results (Table 3), except that the difference between the small and large hospitals in the smaller cohort was no longer significant.

We also set the number of clusters as 4 in the k-means analysis for cohort B. Results were similar to the first analysis, including 1 cluster in which hospitals tended to have low overuse scores across all services except for spinal fusion—the majority of major teaching hospitals were in this cluster (164 of 223 major teaching hospitals [73.5%])—and another cluster where hospitals had high overuse scores for imaging services (eFigure 5 in the Supplement). The proportions of hospital characteristics within each cluster are shown in eTable 4 in the Supplement, with similar findings as cohort A.

## Discussion

To our knowledge, the method of scoring of low-value services reported here represents the first metric that can be applied at a hospital level, allowing for comparisons across hospitals and examination of hospital characteristics associated with low-value care. Our findings that larger hospitals, major teaching hospitals, and nonprofit hospitals are more likely to avoid overuse may provide guidance for targeted improvement efforts. For example, payers such as CMS might consider structuring financial incentives for reducing overuse around specific hospital factors in our data. Our cluster analyses might also point to ways for payers to target incentives for reducing particular types of overuse; diagnostic testing, for example, is already low in major teaching hospitals but higher in others.

We also found regional differences in hospitals’ avoidance of overuse, and CMS could prioritize its efforts by regions. Colla et al^8^ also found their overuse composite measure (at the hospital referral region level) of tests and treatments was highest in the southern US.

We used total numerator volumes to weight the composite overuse score in order to underemphasize services with low volumes, and our conclusions based on the composite score are dependent on this choice. We could have used weights based on the total costs of each of the services, the likely patient harm from each of these services, or how certain the evidence is to avoid a service. Each of these weightings would create an overall score for hospitals based on different judgments about the consequences of delivering low-value services (eg, the value of a low-volume, expensive procedure vs a high-volume, low-cost service).

The cluster analysis revealed underlying patterns of hospital characteristics associated with overuse that were stable within and across the 2 study populations. For example, both cohorts included a cluster where hospitals had high rates of imaging overuse; this could mean many or even all of the hospitals in this cluster share common business practices, culture, or payer mix. This consistency reveals a structure within the data but is hypothesis generating. Further studies will be required to elucidate the factors responsible for these observations.

Within both cohorts A and B, 1 cluster exhibited notably lower overuse scores on all services with the exception of spinal fusion. This cluster had a disproportionate share of larger, metropolitan-area nonprofit teaching hospitals in the northeast. Why this service might be an outlier among these hospitals is unclear. It may be driven by patient demand for spinal fusion, but more likely factors for its entrenchment include the sparsity of high-quality evidence^29^ and such hospital-level factors^30^ as investment in devices, local market competition,^31^ and the procedure’s relatively high profit margin.^32^

### Limitations

This study has some limitations. Clinical details are not always captured in claims data, and indicators of low-value care may underestimate or overestimate true rates.^33^ We used a set of published indicators, some of which are from another overuse index that has external validation against regional costs and outcomes.^13^ In addition, our improvements to *ICD-9*–based claims data algorithms for classifying low-value services enhance the specificity of our results.

Our analysis was based on Medicare fee-for-service claims. There may be different trends of overuse among commercially insured persons, perhaps owing to different policies and coverage or provider reimbursements. At a clinician level, however, both Charlesworth et al^7^ and Colla et al^34^ showed that clinicians did not change their practices regarding provision of low-value services depending on a patient’s insurance (Medicare vs commercially insured). They found, instead, that geography was a bigger driver in variation of low-value service utilization.

Our results do not apply to specialty hospitals, which we defined conservatively as those with more than 20% of orthopedic or cardiac cases. These hospitals may have substantially different rates of overuse than general hospitals.

Although the patterns across hospital characteristics in the smaller group of hospitals in cohort B were similar to those in cohort A, they may not persist in the full population of 3359 hospitals with capacity for at least 1 service. Our findings are also limited by the set of specific low-value services we investigated. Other patterns may emerge when more services are included.

## Conclusions

Results of this cross-sectional study show that measurements of low-value services using Medicare claims data can be applied to individual hospitals to compare their overall rates of overuse. This analysis revealed differences in overuse by hospital characteristics such as teaching status, region, and nonprofit status. This novel measurement of hospital-associated overuse is a useful method for combining results across multiple indicators of overuse and comparing overall overuse within US hospitals.

## Notes

Supplement. **eTable 1.** Details of the Service Capacity Filter Rules for Each Service **eTable 2. **Details of the Low-Value Service Algorithms and Any Modifications **eFigure 1.** Distribution of the Overuse Rates for Hospitals (Cohort A, N = 2,415) **eFigure 2. **Density Plots of the Normalized Overuse Rates and Overuse Score **eFigure 3. **Scree Plot for K-Means Cluster Analysis of (n ≥ 7) Service Overuse Rates **eFigure 4. **Cluster Visualization of Results From K-Means Analysis **eTable 3. **Hospital Characteristics Significantly Associated With K-Means Clusters (Cohort A) **eFigure 5.** Cluster Comparison for Hospitals With Capacity for All Services (N = 1,350 Hospitals) **eTable 4. **Hospital Characteristics Significantly Associated With K-Means Clusters (Cohort B)
