# Error in Abstract and Results

**DOI:** 10.1001/jamanetworkopen.2021.17001

**Published:** 2021-06-04

In the Original Investigation titled “Assessment of Overuse of Medical Tests and Treatments at US Hospitals Using Medicare Claims,”^1^ published April 27, 2021, the number of White patients was written incorrectly in the Abstract and Results as 101 017 191; the correct value is 1 017 191. This article has been corrected.^1^
