# Supplementary material for: Assessment of Overuse of Medical Tests and Treatments at US Hospitals Using Medicare Claims
Source: JAMA Netw Open. 2021 Apr 27;4(4):e218075. doi: 10.1001/jamanetworkopen.2021.8075 (PMC8080218; doi:10.1001/jamanetworkopen.2021.8075)
Supplement: Supplement. — eTable 1. Details of the Service Capacity Filter Rules for Each Service eTable 2. Details of the Low-Value Service Algorithms and Any Modifications eFigure 1. Distribution of the Overuse Rates for Hospitals (Cohort A, N = 2,415) eFigure 2. Density Plots of the Normalized Overuse Rates and Overuse Score eFigure 3. Scree Plot for K-Means Cluster Analysis of (n ≥ 7) Service Overuse Rates eFigure 4. Cluster Visualization of Results From K-Means Analysis eTable 3. Hospital Characteristics Significantly Associated With K-Means Clusters (Cohort A) eFigure 5. Cluster Comparison for Hospitals With Capacity for All Services (N = 1,350 Hospitals) eTable 4. Hospital Characteristics Significantly Associated With K-Means Clusters (Cohort B) [file jamanetwopen-e218075-s001.pdf]

Supplementary Online Content

Chalmers K, Smith P, Garber J, et al. Assessment of overuse of medical tests and treatments at US hospitals using Medicare claims. *JAMA Netw Open*. 2021;4(4):e218075. doi:10.1001/jamanetworkopen.2021.8075

eTable 1. Details of the Service Capacity Filter Rules for Each Service

eTable 2. Details of the Low-Value Service Algorithms and Any Modifications

eFigure 1. Distribution of the Overuse Rates for Hospitals (Cohort A, N = 2,415)

eFigure 2. Density Plots of the Normalized Overuse Rates and Overuse Score

eFigure 3. Scree Plot for K-Means Cluster Analysis of (n ≥ 7) Service Overuse Rates

eFigure 4. Cluster Visualization of Results From K-Means Analysis

eTable 3. Hospital Characteristics Significantly Associated With K-Means Clusters (Cohort A)

eFigure 5. Cluster Comparison for Hospitals With Capacity for All Services (N = 1,350 Hospitals)

eTable 4. Hospital Characteristics Significantly Associated With K-Means Clusters (Cohort B)

This supplementary material has been provided by the authors to give readers additional information about their work.

© 2021 Chalmers K et al. *JAMA Network Open.*

eTable 1. Details of the Service Capacity Filter Rules for Each Service

We used the following rules to decide whether a hospital had the necessary equipment/capacity to carry out a specific service. Hospitals with capacity but zero overuse were true 'zeroes', while hospitals with zero capacity were treated as missing. The capacity filter for hysterectomy, carotid endarterectomy, coronary artery stenting and spinal fusion were the denominator definitions of these services (an annual average of at least one count of the procedure without any condition qualifiers).

**Service**	**Capacity filter rule**
Arthroscopic knee surgery	An annual average (2015-2017) of at least one instance of ICD-9-PCS 80.x in MedPar or outpatient (incision and excision of joint structures), or CPT codes 29877, 29879, 29880, 29881, G0289 in outpatient (specific arthroscopy codes from low-value service algorithm).
Vertebroplasty	An annual average (2015-2017) of at least one instance of ICD-9-PCS 81.x (repair and plastic operations on joint structures), 80.x (incision and excision of joint structures) in MedPar or outpatient, or CPT codes 22520, 22521, 22523, 22524, 22510, 22511, 22512, 22513, 22514, 22515 in outpatient (specific vertebroplasty codes from low-value service algorithm).
Inferior vena cava filter	An annual average (2015-2017) of at least one instance of ICD-9-PCS 38.x (incision, excision, and occlusion of vessels), 88.6x (phlebography) in MedPar or outpatient, or CPT codes 37191, 37192, 75940 in outpatient (specific IVC filter codes from low-value service algorithm).
Renal artery stenting	An annual average (2015-2017) of at least one instance of ICD-9-PCS 88.4x (arteriography using contrast material), 88.5x (angiocardiography using contrast material), 39.50 (angioplasty of other non-coronary vessel) in MedPar or outpatient, or CPT codes 35471, 35450, 37205, 37207, 37236, 75960, 7596 in outpatient (specific stenting codes from low-value service algorithm).
Pulmonary artery catheterization	An annual average (2015-2017) of at least one instance of ICD-9-PCS 89.61, 89.62, 89.63, 89.64 (circulatory monitoring: systemic arterial, central venous, pulmonary artery pressure monitoring, or pulmonary artery wedge monitoring) in MedPar or outpatient, or CPT codes 93503 in outpatient (Swan Ganz placement, specific code from low-value service algorithm).
EEG*	An annual average (2015-2017) of at least one instance of ICD-9-PCS code 89.14 in MedPar or outpatient, or CPT codes 95812, 95813, 95816, 95819, 95822, 95827, 95830, 95957, 3650F, 95812, 95813, 95816, 95819, 95822, 95827 in outpatient (that is, electroencephalogram services).
Carotid artery imaging	An annual average (2015-2017) of at least one instance of ICD-9-PCS code 88.9[1-7] (MRI for head), 88.01, 87.41, 87.03 (CT scan), or 88.7x (head ultrasound) in MedPar or outpatient, or CPT codes 70498, 70547, 70548, 70549, 93880, 93882, 3100F (imaging studies) in outpatient.
Head imaging	An annual average (2015-2017) of at least one annual instance of ICD-9-PCS code 88.9[1-7] (MRI for head), 88.01, 87.41, or 87.03 (CT scan) in MedPar or outpatient, or CPT codes 70450, 70460, 70470, 70551, 70552, 70553 (imaging studies) in outpatient.

*Corresponds to two low-value service metrics: EEG for headache and EEG for syncope.

© 2021 Chalmers K et al. *JAMA Network Open.*

eTable 2. Details of the Low-Value Service Algorithms and Any Modifications

We made the following updates to the published overuse/low-value service indicators of Schwarz and Segal. For services with syncope or headache, we required that this diagnosis is the reason for admission or that there was no history in the claims for this diagnosis. We updated the spinal fusion indicator to exclude cervical spinal fusions, and allowed disc degenerative disorders as a potential indicator of overuse. For coronary stents for stable disease, we used additional exclusion criteria of ICD-9-CM codes for myocardial infarctions and unstable angina within the current claim and any recent claim (14 days), rather than the original specific definition which used the indication date for myocardial infarction provided in the Chronic Conditions Warehouse comorbidity table.

Some of the Schwartz indicators flagged services using the AMA's Current Procedural Terminology (CPT) codes in carrier and outpatient claims, and we instead found these codes used in outpatient and inpatient claims. For coronary stents procedures, we flagged services using CPT codes in outpatient claims, or if the CPT codes in the inpatient table matched (within two days) to ICD-9-CM procedure code '0066: percutaneous transluminal coronary angioplasty' in MedPar claims.

**Service and source**	**Original**	**Updates**
EEG for headache (Schwartz 2015) Specific version	EEG with headache diagnosis in the claim. No epilepsy or convulsions noted in current or prior claims (during the year).	Added requirement for headache to be either 1) primary diagnosis, admitting diagnosis or reason for admission, or 2) no previous headache diagnosis in prior 2 years.
Carotid artery imaging for syncope (Schwartz 2015) Specific version	Carotid imaging with syncope diagnosis. No history of stroke or TIA. No stroke, TIA, or focal neurological symptoms noted in claim.	Added requirement for syncope as either 1) primary diagnosis, admitting diagnosis or reason for admission, or 2) no previous syncope diagnosis in prior 2 years.
Head imaging for syncope (Schwartz 2015) Specific version	CT or MR Imaging of the head with a diagnosis of syncope in the imaging claim. No current diagnosis of epilepsy, stroke/TIA, head trauma, convulsions, altered mental status, nervous system symptoms, speech problems, history of stroke.	Added requirement for syncope as either 1) primary diagnosis, admitting diagnosis or reason for admission, or 2) no previous syncope diagnosis in prior 2 years.
Coronary stenting for stable coronary disease (Schwartz 2015) Sensitive version	Coronary stent placement or balloon angioplasty for patients with an established diagnosis of ischemic heart disease or angina (at least 6 months prior to the procedure). Procedure not associated with an ER visit, which might be indicative of acute coronary syndrome	Added additional exclusion of unstable angina or myocardial infarction in the previous 14 days.
EEG for syncope (Segal 2015)	EEG on the same claim as diagnosis of syncope or at any time during the hospitalization with a code for syncope.	Required syncope as either 1) primary diagnosis, admitting diagnosis or reason for admission, or 2) no previous syncope diagnosis in prior 2 years.

© 2021 Chalmers K et al. *JAMA Network Open.*

Service and source	Original	Updates
Spinal fusion without radicular pain (Segal 2015)	Spinal fusion or laminectomy without symptoms of clearly of herniated disc, radicular pain.	Restricted to cases where MedPar claims had an ICD-9-CM spinal fusion code for the lower back and a DRG for spinal fusion (adding 453, 454, 455 to the original list). Removed disc degeneration and degenerative disc disorder from the original list of exclusions (removing ICD-9-CM 722.51, 722.52, 722.6, 722.90, 722.92, 722.93).
Renal artery angioplasty or stenting (Schwartz 2015). Specific version.	Renal/visceral angioplasty or stent placement. Diagnosis of renal atherosclerosis or renovascular hypertension, and no diagnosis of fibromuscular dysplasia of renal artery, in procedure claim.	-
Carotid endarterectomy for asymptomatic patients (Schwartz 2015). Sensitive version.	Carotid endarterectomy for patients without a history of stroke or TIA and without stroke, TIA, or focal neurological symptoms noted in claim. Operation not associated with an ER visit.	-
Inferior vena cava filters for the prevention of pulmonary embolism (Schwartz 2015). Specific version (sensitive is the same).	Any IVC filter placement.	-
Vertebroplasty or kyphoplasty for osteoporotic vertebral fractures (Schwartz 2015). Sensitive version.	Vertebroplasty/kyphoplasty for vertebral fracture.	-
Arthroscopic surgery for knee osteoarthritis (Schwartz 2015). Sensitive version.	Arthroscopic debridement/ chondroplasty of the knee with diagnosis of osteoarthritis or chondromalacia in the procedure claim.	-
Hysterectomy for benign disease (Segal 2015).	All women with hysterectomy excluding a malignancy diagnosis.	-

© 2021 Chalmers K et al. *JAMA Network Open.*

**Service and source**	**Original**	**Updates**
Pulmonary Artery Catheterization in the ICU (Schwartz 2015. Specific version.	Pulmonary artery catheterization for monitoring purposes during an inpatient stay that involved an ICU but not a surgical DRG. Exclude claims that involved pulmonary hypertension, cardiac tamponade, or preop assessment diagnoses.	-

© 2021 Chalmers K et al. *JAMA Network Open.*

eFigure 1. Distribution of the Overuse Rates for Hospitals (Cohort A, N = 2,415)

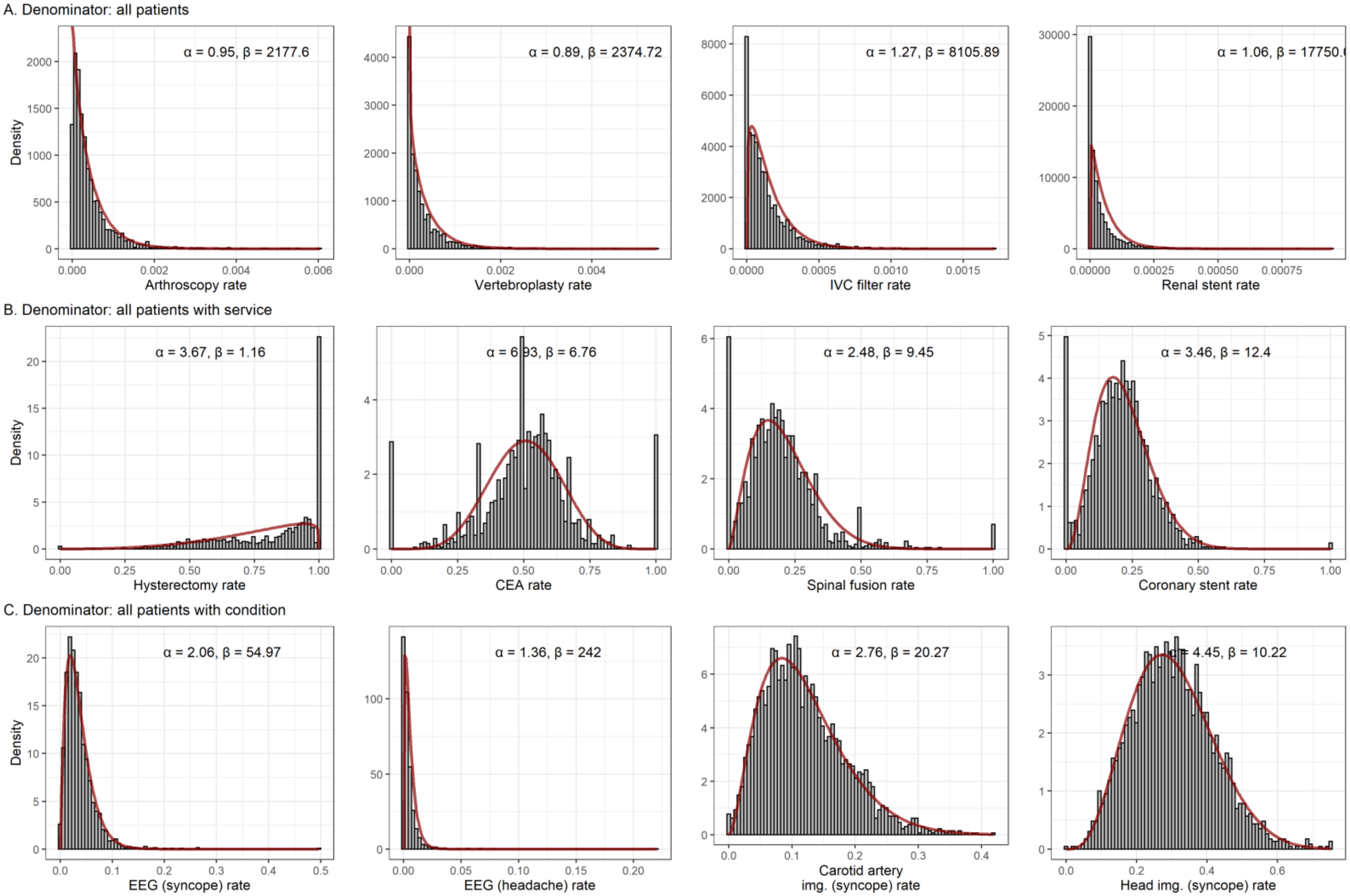

The fitted Beta distribution used for the reliability adjustment of rates is shown using the red line. Overuse ratios are measured as the count of low-value services by either A) all patients at the hospital B) all patients at the hospital with the service and C) all patients at the hospital with the condition. IVC: inferior vena caval filter; CEA: carotid endarterectomy; EEG: electroencephalography; img.: imaging.

© 2021 Chalmers K et al. *JAMA Network Open.*

eFigure 2. Density Plots of the Normalized Overuse Rates and Overuse Score

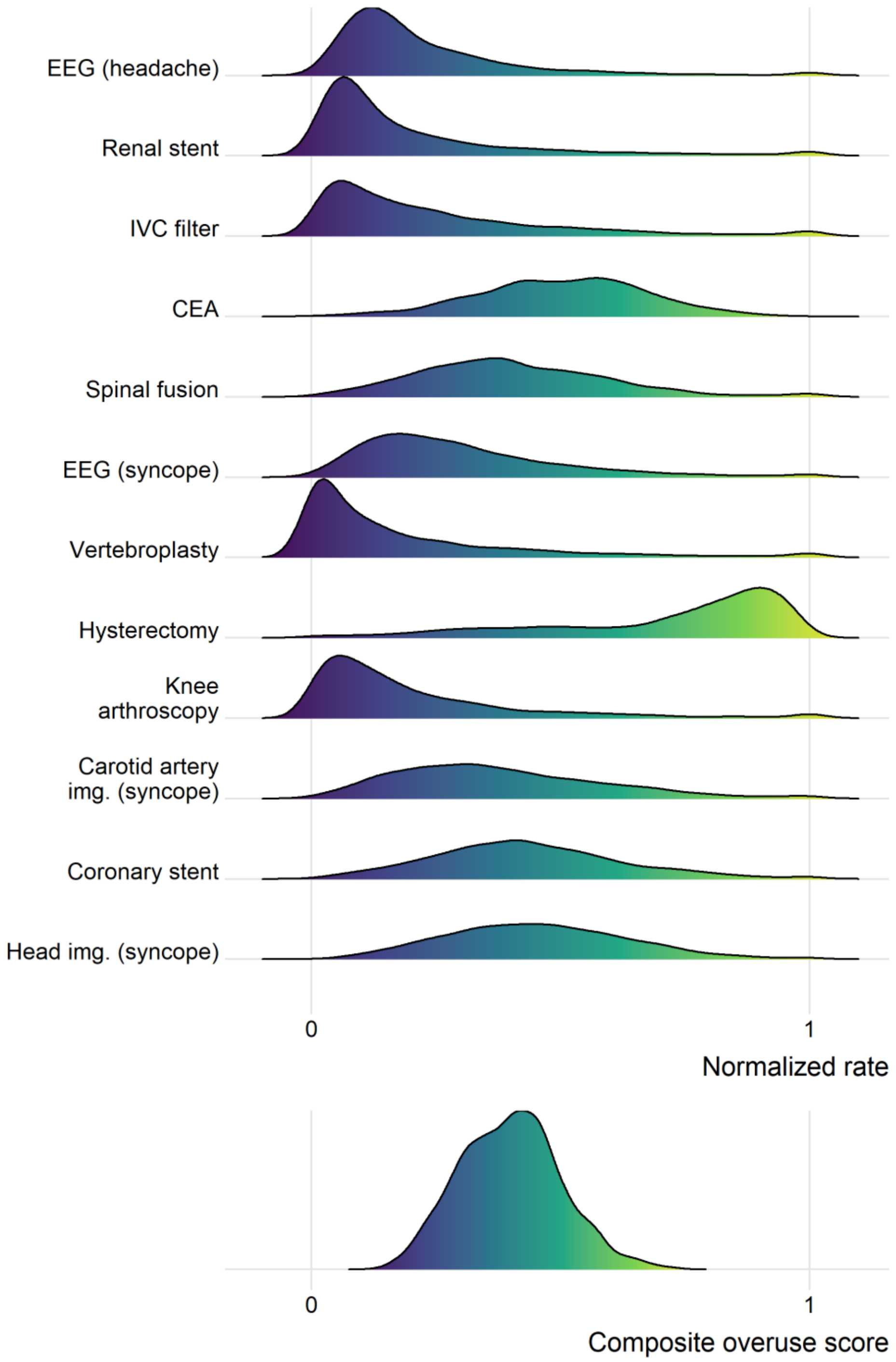

Density plots of Panel A) the normalized service overuse rates across hospitals (cohort A; N=2,415) and Panel B) the composite overuse score, a weighted sum of these rates. Services in panel A are ordered from the lowest to highest contribution in the weighted sum (that is, EEG for headache rates have the lowest weighting and head imaging for syncope has the greatest weighting in the composite). IVC: inferior vena caval filter; CEA: carotid endarterectomy; EEG: electroencephalography; img.: imaging.

© 2021 Chalmers K et al. *JAMA Network Open*.

eFigure 3. Scree-Plot for K-Means Cluster Analysis of (n ≥ 7) Service Overuse Rates

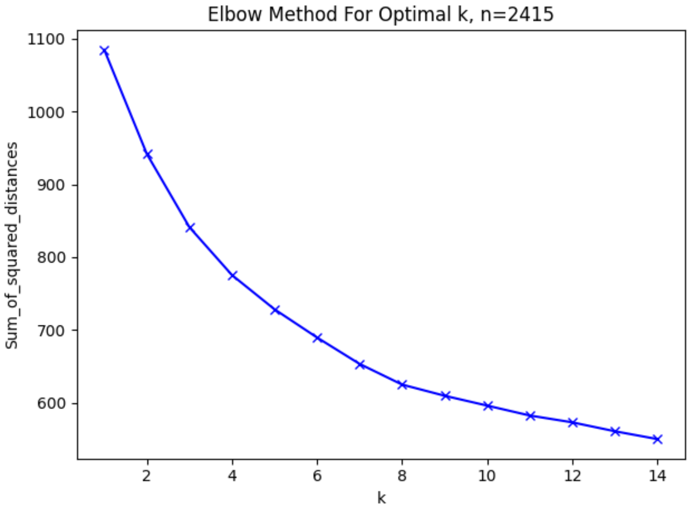

The scree-plot for the K-means cluster analysis. K is the number of clusters, while the y-axis shows the sum of squared distances between the points in each cluster.

eFigure 4. Cluster Visualization of Results From K-Means Analysis

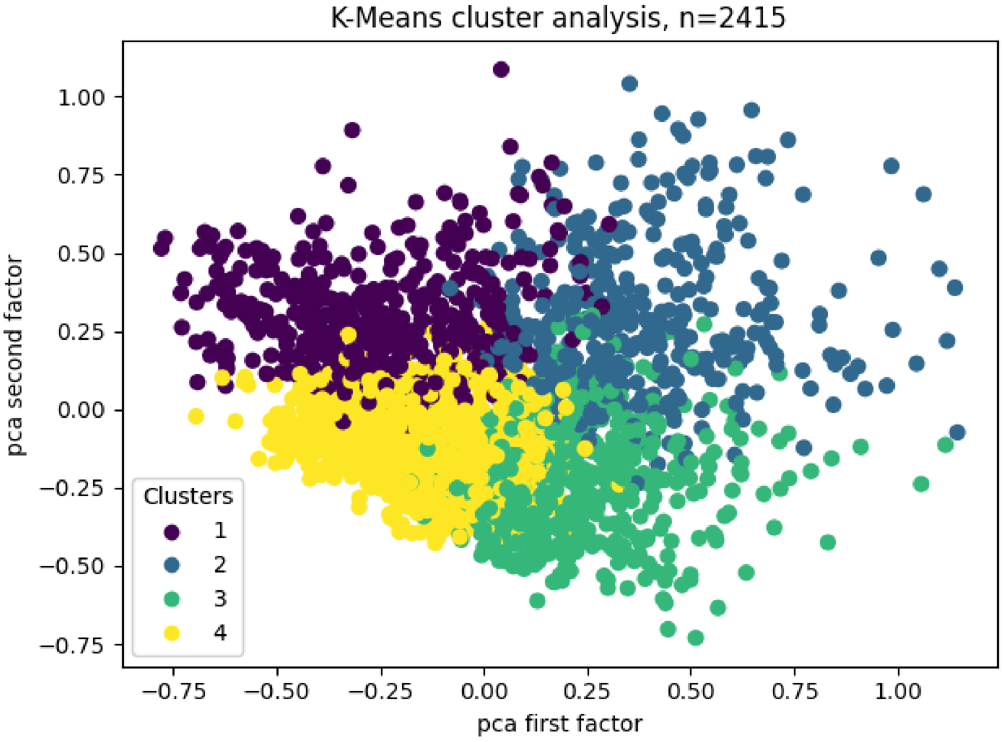

Visualization of the four clusters in two dimensions, which are the first and second factors from a principal components analysis (PCA) applied to the thirteen overuse scores (note these first two factors explain 37.39% of the variability in the data).

© 2021 Chalmers K et al. *JAMA Network Open.*

eTable 3. Hospital Characteristics Significantly Associated With K-Means Clusters (Cohort A)

Cluster	Hospital characteristic	Within cluster (%, N) (a)	Entire cohort (%, N) (b)	Difference (%) (a-b)
1: Low overuse, except high spinal fusion overuse (N = 454)	Major teaching	41.2 (162)	16.0 (216)	25.2
	200+ bed size	92.6 (364)	75 (1,013)	17.6
	Non-profit	92.9 (365)	81.5 (1,100)	11.4
	Non-teaching	19.3 (76)	45.8 (618)	-26.4
	Metro area	98 (385)	93.1 (1,257)	4.9
	South	25.4 (100)	39.9 (539)	-14.5
	Northeast	25.7 (101)	16.7 (226)	9.0
	6-199 bed size	7.4 (29)	25.0 (337)	-17.6
2: High procedure overuse (N = 400)	South	61.2 (197)	39.9 (539)	21.3
	Non-profit	64.3 (207)	81.5 (1,100)	-17.2
	For profit	35.7 (115)	18.5 (250)	17.2
	Non-teaching	57.1 (184)	45.8 (618)	11.4
3: High diagnostic services overuse (N = 515)	Non-teaching	59.8 (156)	45.8 (618)	14.0
	200+ bed size	64.8 (169)	75.0 (1,013)	-10.3
	6-199 bed size	35.2 (92)	25.0 (337)	10.3
4: High hysterectomy overuse (N = 1,046)	200+ bed size	59.6 (223)	75.0 (1,013)	-15.4
	6-199 bed size	40.4 (151)	25.0 (337)	15.4

Hospital characteristics are reported if the within cluster proportion is largely different from the overall cohort proportion. Rows are ordered by the most significant hospital characteristic within each cluster. For a hospital characteristic/cluster comparison to be included in the table, this difference was significant at a 5% significance level and the effect size was non-trivial (Cohen's *h* value was greater than 0.2). The effect size criteria was included as the p-value from the significance test is sensitive to large sample sizes.

© 2021 Chalmers K et al. *JAMA Network Open.*

eFigure 5. Cluster Comparison for Hospitals With Capacity for All Services (N = 1,350 Hospitals)

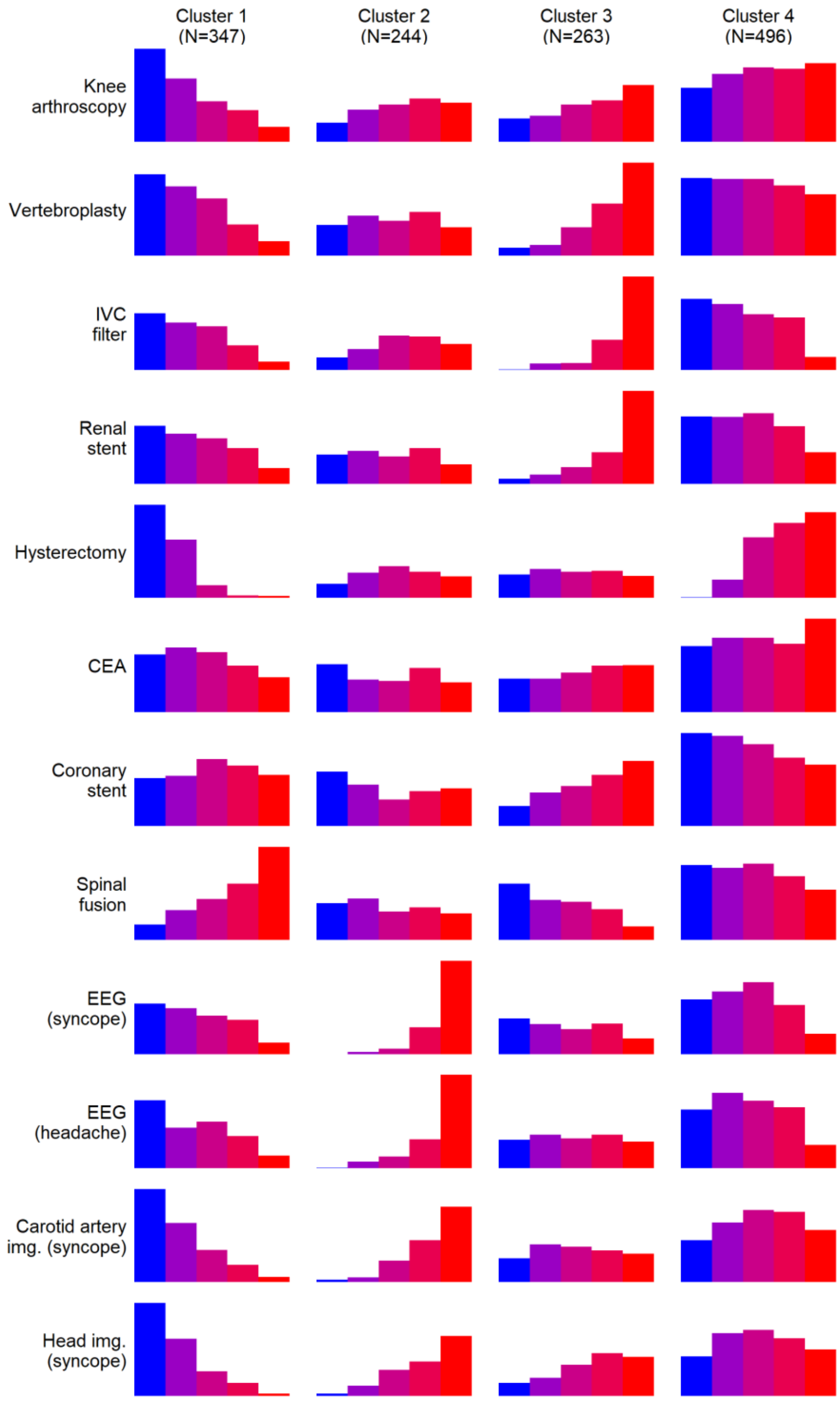

Bars show the relative counts of overuse scores (blue: lowest quintile of adjusted overuse ratios to red: highest quintile of adjusted overuse ratios) for each service across the four clusters. IVC: inferior vena cava; CEA: carotid endarterectomy; EEG: electroencephalography; img.: imaging.

eTable 4. Hospital Characteristics Significantly Associated With K-Means Clusters (Cohort B)

Cluster	Hospital characteristic	Within cluster (%, N) (a)	All cohort (%, N) (b)	Difference (a – b)
1: Low overuse, except spinal fusion (347 hospitals)	Major teaching	46.4 (161)	16 (216)	30.4
	200+ bed size	91.6 (318)	75 (1,013)	16.6
	NP	94.8 (329)	81.5 (1,100)	13.3
	Non-teaching	17.6 (61)	45.8 (618)	-28.2
	Metro	97.4 (338)	93.1 (1,257)	4.3
	South	23.6 (82)	39.9 (539)	-16.3
	6-199 bed size	8.4 (29)	25 (337)	-16.6
2: High diagnostic services overuse (244 hospitals)	Non-teaching	57.4 (140)	45.8 (618)	11.6
3: High procedure overuse (263 hospitals)	NP	62.7 (165)	81.5 (1,100)	-18.7
	FP	37.3 (98)	18.5 (250)	18.7
	South	59.3 (156)	39.9 (539)	19.4
4: High hysterectomy overuse (496 hospitals)	6-199 bed size	38.5 (191)	25 (337)	13.5
	200+ bed size	61.5 (305)	75 (1013)	-13.5
	Non-teaching	57.1 (283)	45.8 (618)	11.3

Hospital characteristics are reported if the within cluster proportion is largely different from the overall cohort proportion. Rows are ordered by the most significant hospital characteristic within each cluster. For a hospital characteristic/cluster comparison to be included in the table, this difference was significant at a 5% significance level and the effect size was non-trivial (Cohen's h value was greater than 0.2). The effect size criteria was included as the p-value from the significance test is sensitive to large sample sizes.

© 2021 Chalmers K et al. *JAMA Network Open.*
